# Concerning Various Methods Advocated for Obviating the Necessity of Extracting Devitalized Tooth-Pulps

**Published:** 1893-12

**Authors:** W. D. Miller

**Affiliations:** Berlin, Germany.


ARTICLE VI.

### CONCERNING VARIOUS METHODS ADVOCATED FOR OBVIATING THE NECESSITY OF EXTRACTING DEVITALIZED TOOTH-PULPS.

BY DR. W. D. MILLER, BERLIN, GERMANY.

Read at World's Columbian Dental Congress.

The practice now in vogue among good practitioners, of thoroughly removing the pulp and filling the root-canal to the apex, is usually so easily carried out in the incisors and cuspids, and gives such sure results, that there is no probability that a better method will ever be found. But when we extend this treatment to the bicuspids and molars, the labor and expense entailed are frequently so great as to put it beyond the reach of the great majority of the human race, and the method is not always successful. It will consequently be a great boon if some means or method can be devised which would render unnecessary the removing of the pulp and filling the root-canals of molars.

While every dentist has now and then knowingly left remains of the pulp in narrow and tortuous canals, or in canals obstructed by calcific matter, and while many dentists in Europe have contented themselves with simply devitalizing the pulp, filling over it with amalgam and *leaving the root to nature*, the first *systematic* attempt to do away entirely with the necessity of extracting the root-portions of the pulp appears to have been made by Witzel, who, in 1874, presented the view that an application of arsenious acid carefully made to the inflamed pulp devitalized only the disease tissue, and that by amputating the coronal portion of the pulp twenty-four hours after the application, the ends of the root-stumps might be treated as healthy, freshly-exposed pulps.

Dr. Miller then presented briefly the methods devised by Witzel, Baume and Herbst, the latter as put forth by its author and as modified by Bodecker, and summarized their advantages and disadvantages. Continuing, he said:

Perhaps the majority of dentists have also made more or less extensive use of the method recommended by Bodecker, when they have left a portion or the whole of the pulp in the buccal roots of upper, or mesial root of lower molars, and filled directly over them, after thoroughly bathing them with carbolic acid or some other antiseptic.

I have for a long time felt that the solution of the problem was to be sought for in the direction pointed out by Witzel, except that our efforts should be directed, not to retaining the vitality of the root stumps, but to preventing their subsequent decomposition by impregnating them with a suitable antiseptic. I am convinced that the success of the impregnation method depends, to a very great extent, upon the character of the antiseptic employed, and and upon its chemical action upon the pulp apart from its antiseptic action.

The qualities desirable appear to me to be:

1. It must be a strong antiseptic.

2. It must be sufficiently soluble and diffusible to guarantee the impregnation of the whole pulp.

3. It must not be so diffusible that it will be completely taken up by the surrounding tissue and finally disappear together, as is the case with applications of carbolic acid. It is my impression that there is greater danger in too great solubility than in insolubility.

4. A coagulating action upon the tissue of the pulp appears desirable, though not absolutely essential. A pulp which is coagulated into a hard, insoluble body, is less likely to furnish nourishment for bacteria and offer irritation to the periapical tissue than one in a soft or semiliquid condition. One cause of the failure of Baume's borax treatment is probably the conversion of the pulp into a liquid, or semi-liquid, soapy mass, with a strong alkaline smell and reaction, which can hardly be indifferent to the tissue about the apical foramen.

5. It is desirable that the substance employed have no irritating action upon the pericementum.

6. It should not discolor the tooth, although, as the treatment concerns chiefly molars, a slight discoloration need not be considered as a very serious matter.

7. Solid substances are better adapted to the purpose than liquids.

It is difficult to find a substance which fulfils all the above mentioned conditions.

According to the results obtained from over five hundred experiments, I have divided dental antiseptics into three groups:

1. Those possessing in a high degree the power of imparting antiseptic qualities to root-pulps, such as cyanide of mercury, bichlorid of mercury, diaphtherin, sulphate of copper, salicylate of mercury, oil of cinnamon, ortho-kresol, carbolic acid, trichlor-phenol, chlorid of zinc. The last four are, however, decidedly inferior to the others; they penetrate the pulp very rapidly, chlorid of zinc surprisingly so, but they are lacking in the necessary powerful antiseptic qualities, and are so diffusible that in the course of a few weeks they disappear altogether from the pulp.

2. Those of doubtful value: Thymol, salicylic acid, eugenol, campho-phenique, hydronaphthol, A and B naphthol, aceticotartrate of aluminum and some essential oils, resorcin, thallin, sulpho-carbolate of zinc, oil of birch, iodid of sodium, nitrate of sodium, etc.

3. Those nearly or quite worthless: Iodoform, basic anilin coloring-matters, borax, boracic acid, dermatol, europhen, chlorid of lime, peroxid of hydrogen, sozoiodol salts, iodol, tincture of iodin, spirits of camphor, naphthalin, etc.

The attempt to apply these results to practice was first made with the bichloride of mercury, which has been used since 1890 in some four hundred to five hundred cases, first in the form of small tablets, having the composition:

Sublimate, 0.01 gram; Sublimate, 0.01; or

Boracic acid, 0.02 gram; Common salt, 0.02.

The pulp having been completely devitalized, the pulp-chamber was thoroughly opened and cleansed, and a tablet applied and slightly crushed with an amalgam plugger, moistened with water and covered with a layer of tin foil (I now use gold foil), and the amalgam or cement filling immediately inserted. In about thirty per cent. of the cases severe pain occurred on the day following the application, and on account of this disagreeable symptom these tablets were abandoned and the following substituted:

Sublimate, 0.0075 gram;
Thymol, 0.0075 "

These are applied in the same manner. The thymol being chiefly designed to prevent the sublimate being so rapidly absorbed, besides giving a greater permanency to the application, by reducing its solubility. Very seldom, so far, has pain followed the use of these tablets, while experiments out of the mouth show that they still possess sufficient penetrating power.

Another combination employed is:

Sublimate, 0 005 gram;
Thymol, 0.005 "
Tannin, 0.005 "

This combination is somewhat empirical, though the design of the tannin will be apparent to every one. The combination does not penetrate as rapidly as No. 2, and discolors the tooth more.

Cyanide of mercury has also been employed in combination with thymol, in the following form:

Cyanide of mercury, 0.0075 gram.
Thymol, 0.0075 "

Also the salicylate of mercury in the same form. This I think deserving of a trial. Its sparing solubility justifies the belief that its action will be more permanent than that of sublimate. The sulphate of copper may be used in pure form, but it naturally causes serious discoloration of the tooth at the neck, and is also, I fear, too soluble to give permanent results, in pure form. More recently, I have directed my experiments toward the discovery of some substance which possesses the desired qualities without discoloring the tooth. Thus far I have obtained the best results from diaphtherin (oxychinaseptol), an antiseptic recently introduced by Emmerich. It may be applied in pure form. Among liquid antiseptics, the oil of cinnamon takes the first place, and I have much faith in its power to conserve the dead pulp. Like all the liquids, however, it is difficult to apply, and has, besides, the disagreeable quality of discoloring the tooth yellowish-brown. The combinations which I have chiefly employed is that of sublimate and thymol. (I have not had opportunity to sufficiently test the others in practice, though I am now using, by way of experiment, the salicylate, and, to some extent, the cyanide of mercury.) It has been employed at the Dental Institute of the University of Berlin in over two hundred cases. Of these, only one failure has come to my knowledge.

Time is the only test for methods like those under consideration, and we can scarcely expect to arrive at a definite conclusion in less than five to ten years. Nor should we be hasty in the application of methods of this nature. One or two cases every month, at least for the first year or two, is all that a careful dentist ought to risk in private practice. Cases should be chosen which are very difficult to treat, and which are otherwise frequently treated by the forceps, such as distal cavities of second or third molars, buccal cavities of third molars, etc. It is not possible at present to form a reliable estimate as to the value of this method of treating teeth; it may also be that much better materials will be found for the purpose than those suggestsd above. There are, at least, reasons for believing that by a careful application of this method, many teeth may be saved which otherwise would be sacrificed to the forceps, or, what is much worse, be allowed to crumble away.

[The President exhibited two small bottles containing the preparations which Dr. Miller had recommended, and passed them around for inspection.]

DISCUSSION.

Dr. Frank Abbott (New York City).—I take entirely different views of this matter from the author of the paper and the gentlemen who have been quoted. The only one condition where I think of using any material for devitalizing the pulps of teeth, is where it is impossible to stop pain. I have, perhaps, in the last fifteen years used arsenic in teeth as many as three or four times, and no more. To detail to you how I avoid using arsenic and keep my patient along in a comfortable condition would be comparatively a large story. The line of treatment after devitalization, or of a tooth with a dead pulp, is a question of more importance, apparently, as borne upon by this paper, than any other.

For a number of years I have had a practice that seems from what has been said in reference to it, to be rather unique. I never depend upon the application of an antiseptic in the roots of teeth, but upon a material which I force in and around such, with which is combined an antiseptic strong enough to answer the purpose, and virtually mummify all the material that is left in the canals of the tooth by its action. It surrounds and covers it over, and whatever portion of the pulp is left behind is penetrated by the action of the chlorid of zinc and bichlorid of mercury that is mixed with it. Of course, if the pulps die, they die of their own accord. I have many dead teeth to handle and many to treat in my practice, as everyone has who is in full practice, and I treat them all in one general way. That way is to open the pulp-chamber as carefully as I can, so that I may cleanse it thoroughly of every particle and get thoroughly into all the root-canals. I then, with a very fine gold-pointed syringe, use a 1 in 10,000 solution of bichloride of mercury a grain of bichlorid of mercury in twenty ounces of water —and syringe out these canals just as thoroughly as I can; I then, with a broach or small instrument, penetrate into the canals as far as I can go, stir up the contents, and then wash again, repeating this until I am pretty sure that everything is clean, so that the substance coming out of the tooth as it strikes a white napkin will show a white, clean color instead of staining, as when the canal is filled with dead material. When it is washed thoroughly clean, I fill with oxychlorid of zinc, in which I put a drop of a solution of 1 in 2,000 of bichlorid of mercury, thus combining the antiseptic properties of the bichlorid of mercury and the penetrating and antiseptic properties of the chlorid of mercury and the penetrating and antiseptic properties of the chlorid of zinc and oxid of zinc.

This is the material that mummifies or holds this substance that is left in the roots of the teeth, leaving it in a condition to give no trouble; and it may astonish some of you to know that instead of opening a tooth and treating it day after day for a week or more, I open a tooth and fill it at the same sitting always, unless I have periosteal irritation—soreness of the tooth as I touch it. The crown of the tooth is filled with gold, or any substance that I choose to use, of course, and I dismiss the patient after painting the gums carefully over with a solution of concentrated tincture of aconite root and tincture of iodin. That I always do before my patient leaves the chair. It is a powerful counter-irritant, and does the work of relieving the pressure around the root of the tooth. This, to me, is the simplest, easiest, and most quiet way of getting along with that kind of teeth.

It is the decomposition of the canal contents, and the gases accumulating from that decomposition all the time forcing themselves into the pulp-canal that cause the pain in such cases; the gases cannot get outside, because the cement upon the surface of the root is living tissue, consequently all openings into the structure are closed to the escape of gas, except that which would be taken up in the circulation. In the other way, the opening is there, so that all the gases pass into this pulp-canal.

In the substances that we use for root-filling, we must bear this in mind, that the results of decomposition are what we have to deal with, not the decomposition itself.

Dr. George Cunningham (Cambridge, England).—It is now some years ago since I had the opportunity of knowing what Prof. Miller was doing, and of employing some of these tabloids. So far as cases of this kind are concerded, they are limited, as Dr. Abbott has said. I have no doubt there is a certain percentage of failures. I am acquainted with Dr. Herbst's method of treatment, and I do not believe in his system of hermetic sealing. I support Prof. Miller's statement, which I believe is right, that we can get as good hermetic sealing by his process as by tin in the cavity.

I have tried the Herbst system with so-called "cobalt." Dr. Herbst kindly sent some to me, and my colleague, an eminent chemist, after examining it, said: "In that bottle you have enough arsenic to kill the whole British nation." Prof. Miller delivered an introductory course of lectures on operative dentistry, and showed these experiments in retaining the pulps alive by the cupric and sulphate method. I have used that method in wisdom-teeth. Of course, the alternative treatment is the forceps. If we could find for poor people some means which would shorten the treatment, I trust it will be the practice as used by Dr. Abbott, which will give the opportunity to fill at one sitting. I think the paper we have had to-day is of very great importance, because it has pointed out one way that we can bring our operations within the reach of larger numbers of the community.

Dr. Schreier (of Vienna) addressed the Congress in German, and it was translated by Dr. Ottofy, as follows:

It is indifferent what antiseptic is used; each one leads to the same result. It is only necessary to find material which is easily applicable. If anyone says that he can take an antiseptic material and inject it into the fine canals, it is a matter which is impossible to comprehend. It is necessary that the material should be one which is readily introduced into the root-canal, and whose effect is prompt and immediate. Such a material Dr. Miller did not mention in his essay, but I have published a material of this character—potassium-sodium—which, on another occasion, I will present to the members of the Congress.—*Dental Review.*

